# Clinical Distribution and Molecular Basis of Traditional Chinese Medicine *ZHENG* in Cancer

**DOI:** 10.1155/2012/783923

**Published:** 2012-07-04

**Authors:** Zhen Chen, Peng Wang

**Affiliations:** ^1^Department of Integrative Oncology, Fudan University Shanghai Cancer Center, Shanghai 200032, China; ^2^Department of Oncology, Shanghai Medical College, Fudan University, Shanghai 200032, China

## Abstract

In traditional Chinese medicine (TCM) clinical practice, *ZHENG* (also known as syndrome) helps to guide design of individualized treatment strategies. In this study, we investigated the clinical use of *ZHENG* in TCM-treated cancer patients by systematically analyzing data from all relevant reports in the Chinese-language scientific literature. We aimed to determine the clinical *ZHENG* distributions in six common cancers (lung, liver, gastric, breast, colorectal, and pancreatic) with the expectation of uncovering a theoretical basis for TCM *ZHENG* as a clinical cancer treatment. In addition, we also reviewed the molecular basis underlying *Xue-Yu* (blood stasis), *Shi-Re* (dampness-heat), *Yin-Xu* (Yin deficiency), and *Pi-Xu* (spleen deficiency) *ZHENG* that are commonly found in cancer patients. The results from our summary study provide insights into the potential utility of TCM *ZHENG* and may contribute to a better understanding of the molecular basis of TCM *ZHENG* in cancer.

## 1. Introduction

Traditional Chinese medicine (TCM) has been practiced and recorded in the medical literature for thousands of years. It is considered unique among the world's ancient traditional medicines based upon its integrative use of physiological and holistic theories; for example, a key aim of TCM is to regulate and maintain proper body functions by modulating and exploiting interactions between the patient and their environment. The rich history of TCM has prompted a recent surge in clinical research efforts to evaluate its efficacy as an alternative strategy to the largely pharmaceutical-based approaches used in developed countries to prevent and treat many types of disease, including cancers.

It has been reported that over 90% of modern Chinese cancer patients received some form of TCM during their treatment regimen [1]. The rates of TCM used by health care providers and interest by patients outside of China are continuing or rise annually, especially within the field of oncology [2]. Application of TCM as an adjuvant cancer therapy has been reported to enhance the efficacy of both chemo- and radiotherapy and to help reduce adverse effects of each [3, 4]. Furthermore, the Chinese herbal medicines used in TCM have recently been recognized as an important source for novel drug development, including anticancer drugs [5]. Therefore, western medicine practitioners and researchers are, now more than ever, open to exploring the potential of TCM to enhance conventional treatment of cancer patients [6].

The concept of *ZHENG* occupies an important position in the TCM system and is key to recognizing a patient's disease state and developing an effective, individualized treatment strategy. *ZHENG* is a kind of pathology of the disease development of a body in a certain stage, including the disease wherefrom, the cause, the feature, and the conflicts between healthy energy and evils. It reflects the nature of pathological change at a certain stage and reveals the intrinsic quality of disease more completely, profoundly, and accurately than symptoms. Therefore, the diagnosis of TCM *ZHENG* is to differentiate a disease by analyzing and synthesizing the information, symptoms, and patients' physical status collected through four types of diagnostic methods: inspection, auscultation and olfaction, inquiry, and palpation. According to the combination of diagnostic methods used, different types of *ZHENG* are possible for a single disease, and all may be equally effective. This feature provides flexibility and ready diversification to the disease-targeting therapy, allowing for the treating clinician to take advantage of the patient's personality and mental and spiritual desires to achieve high rates of compliance and completion. Therefore, TCM *ZHENG* differentiation must also be applied to the new TCM efforts being used in cancer patients worldwide. 

The purpose of this study was to identify the clinical usage of* ZHENG* TCM in Chinese cancer patients by systematically searching the relevant Chinese-language medical and scientific literature collections. After analyzing the clinical distribution, the molecular basis underlying TCM *ZHENG* was considered in an attempt to better understand its usefulness in future clinical practice. 

## 2. Literature Search for Publications on TCM *ZHENG *in Chinese Cancer Patients

We searched the four major electronic databases of Chinese-language medical and scientific literature (China National Knowledge Infrastructure (CNKI), Chinese Scientific Journal Database (VIP), Wanfang Database, and Chinese BioMedical Literature Database (CBM)) for publications between 2000 and 2011 that were related to “Zhong Yi” (traditional Chinese medicine), “*ZHENG,*” and “Ai” (cancer). More than 20,000 papers on TCM *ZHENG* in cancer were initially identified and included clinical observations, individual or small-scale case reports, large-scale clinical experiences, and experimental animal studies. 

## 3. Investigation of TCM *ZHENG* in Cancer Patients by Publication Year and Cancer Type

**Figure 1:**
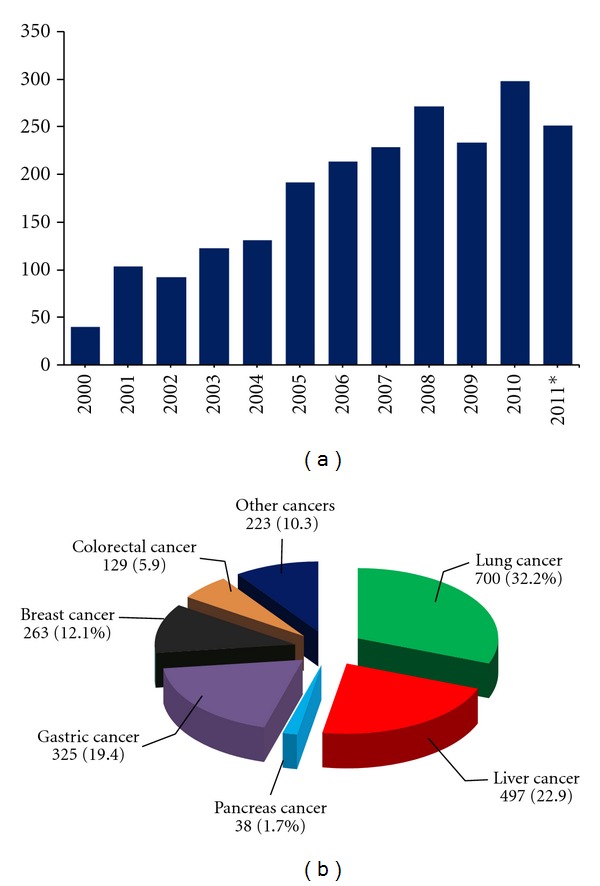
Annual publications on TCM *ZHENG* in cancer. (a) A total of 2175 papers were retrieved by searching the terms “Zhong Yi” (traditional Chinese medicine), “*ZHENG,*” and “Ai” (cancer) through the main Chinese electronic databases, including China National Knowledge Infrastructure (CNKI), Chinese Scientific Journal Database (VIP), Wanfang Database, and Chinese BioMedical Literature Database (CBM), and then analyzed by calculating the annual publications from January 1, 2000 to November 13, 2011. (b) The distributions of cancer types among all the publications. *To November 13, 2011.

As shown in Figure 1, there was a dramatic increase in the number of annual publications of TCM *ZHENG* in cancer patients during the past ten years. Among these articles, 32.2% (700 out of 2175) were related to lung cancer, 22.9% to liver cancer, 19.4% to gastric cancer, 12.1% to breast cancer, 5.9% to colon cancer, 1.7% to pancreatic cancer, and 10.3% to a variety of other types of cancer. This cancer type distribution is consistent with the incidence of cancers in China. It has been reported that the four most frequently diagnosed cancers in Chinese men over the past ten years involved lung, stomach, liver, and colon; Chinese women, however, were most frequently diagnosed with cancers of the breast, lung, stomach, and colon. The incidence of pancreatic cancer in Chinese men and women ranked 8th and 9th, respectively, but produced high mortality (nearly equal to incidence) in both sexes. This result suggests that TCM has been widely applied, as at least one form of treatment, for Chinese cancer patients in modern medical practice. Furthermore, the practice of TCM *ZHENG* in cancer patients has increased steadily over the past decade. 

## 4. Clinical Distributions of TCM *ZHENG *in Chinese Patients with Common Cancers

**Figure 2:**
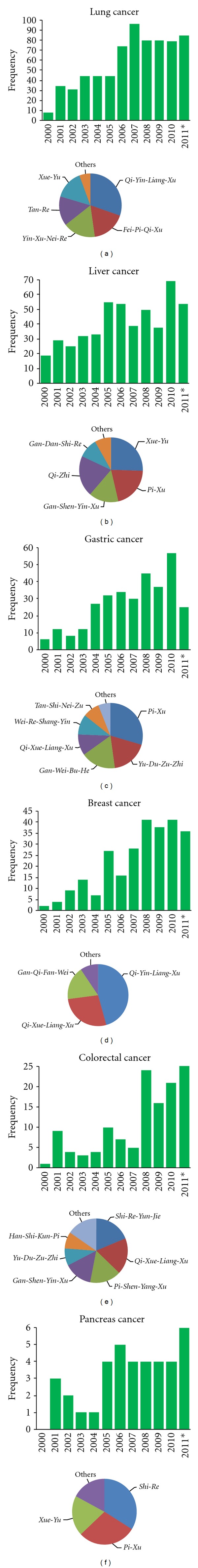
Clinical distributions of TCM *ZHENG* in common cancers. Annual publications for each common cancer were calculated and presented as a histogram. Publications involved with clinical trials and case series, where information on more than 10 cases with *ZHENG* description was available, were further selected. Thirty-two articles reported on lung cancer, 26 on liver cancer, 19 on gastric cancer, 21 on breast cancer, 29 on colorectal cancer, and 17 on pancreatic cancer. Finally, for each type of cancer, the distribution frequency of *ZHENG* was calculated and presented in pie chart. Note: *Qi*-*Yin-Liang-Xu*, deficiency of both *Qi* and *Yin; Fei-Pi-Qi-Xu*, lung-spleen *Qi* deficiency; *Yin-Xu-Nei-Re*, *Yin* asthenia and internal heat; *Tan-Re*, phlegm-heat; *Xue-Yu*, blood stasis; *Pi-Xu*, spleen deficiency; *Gan-Shen-Yin-Xu*, liver-kidney *Yin* deficiency; *Qi-Zhi*, *Qi* stagnation; *Gan-Dan-Shi-Re*, liver-gallbladder dampness-heat; *Yu-Du-Zu-Zhi*, stagnation of blood stasis and toxin; Gan-Wei-Bu-He, liver-stomach disharmony; *Qi-Xue-Liang-Xu*, deficiency of both *Qi* and blood; *Yin-Xu-Nei-Re*, Yin deficiency due to stomach heat; *Tan-Shi-Nei-Zu*, stagnation of phlegm-dampness; *Gan-Qi-Fan-Wei*, liver *Qi* invading stomach; Shi-Re-*Yun-Jie*, stagnation of dampness-heat; *Pi-Shen-Yang-Xu*, asthenic splenonephro-yang; *Yu-Du-Nei-Zu*, stagnation of blood stasis and toxin; *Han-Shi-Kun-Pi*, cold-dampness disturbing spleen.

The six most common types of cancer reported in the studies included in this summary analysis were lung, liver, gastric, breast, colorectal, and pancreatic—collectively accounting for 89.7% of all the publications. We attempted to systematically identify and analyze the clinical *ZHENG* distribution in these six types of cancer. We searched the collection of initially identified relevant studies to identify clinical trials and case series that provided information on ≥10 cases with *ZHENG* description. A total of 144 articles were selected for clinical distribution analysis. The annual distribution frequencies of TCM *ZHENG* for each type of cancer were calculated. The cancer types with *ZHENG* frequency over 10% are presented in Figure 2.

The number of publications describing TCM *ZHENG* in lung cancer increased dramatically from the year 2000 (*n* = 8, in total) to the end of 2011 (*n* = 85, in total). Among these publications, 32 reported results from clinical trials or case series with *ZHENG*-based TCM. Summary analysis indicated that *Qi-Yin-Liang-Xu*, *Fei-Pi-Qi-Xu*, *Yin-Xu-Nei-Re*, *Qi-Zhi-Xue-Yu,* and *Tan-Re* were the most common *ZHENGs* in lung cancer (Figure 2). The number of publications describing TCM *ZHENG* in other types of cancer (liver, gastric, breast, colorectal, and pancreas) also increased dramatically over the past decade. As shown in Figure 2, by the end of 2011, a total of 26 articles had reported data on TCM *ZHENG* in liver cancer, 19 on gastric cancer, 21 on breast cancer, 29 on colorectal cancer, and 17 on pancreatic cancer. The frequency distribution of *ZHENG* for each of these types of cancer was calculated. The results indicated that the main *ZHENGs* for liver cancer were *Xue-Yu*, *Pi-Xu*, *Gan-Shen-Yin-Xu*, *Qi-Zhi*, and *Gan-Dan-Shi-Re*, which accounted for 94.3% of the total. The main *ZHENGs* for gastric cancer were *Pi-Xu*, *Yu-Du-Zu-Zhi*, *Gan-Wei-Bu-He*, *Qi-Xue-Liang-Xu*, *Tan-Shi*, and *Wei-Re-Shang-Yin*, which accounted for 93.9% of the total. The main *ZHENGs* for breast cancer were *Qi-Yin-Liang-Xu*, *Qi-Xue-Liang-Xu*, and *Gan-Qi-Fan-Wei*, which accounted for 90.5% of the total. The main *ZHENGs* for colorectal cancer were *Shi-Re-Yun-Jie*, *Qi-Xue-Liang-Xu*, *Pi-Shen-Yang-Xu*, *Yu-Du-Zu-Zhi*, *Gan-Shen-Yin-Xu*, and *Han-Shi-Kun-Pi*, which accounted for 84.5% of the total. The main *ZHENGs* for pancreatic cancer were *Shi-Re*, *Pi-Xu*, and *Xue-Yu*, which accounted for 82.8% of the total.

## 5. The Molecular Basis for Common *ZHENGs *in Cancer

### 5.1. *Xue-Yu ZHENG* (Blood Stasis)

*Xue-Yu ZHENG* is one of the common syndromes in TCM, characterized by cyanosis (of skin, lips, nails, and/or tongue), ecchymosis and petechia, and irregular pulse (detected by palpation as thin, unsmooth, deep, taut, knotted, slow, or intermittent). In addition, other common clinical signs include blackish complexion, dry skin, and purpura. The *Xue-Yu* status was recently shown to be related with changes of hemorheological properties, such as high-blood viscosity, increased erythrocyte aggregation, increased blood sedimentation, decreased erythrocyte deformability, and decreased hematocrit [7]. 

*Xue-Yu* is associated with many diseases, including cancer. Epidemiological investigation has revealed that *Xue-Yu* is one of the most prominent *ZHENGs* in patients with cancer, especially for those with liver, lung, and pancreatic cancer; the results from our summary analysis agreed with this reported pattern (Figure 2). TCM treatment of cancer patients with *Xue-Yu* using traditional Chinese herbs has shown satisfactory efficacy in clinical practice in China. Since 1990, several retrospective clinical studies have reported strong statistical correlation between tumor metastasis and *Xue-Yu ZHENG*; treating or controlling tumor metastasis, while *Huo-Xue-Hua-Yu (promoting blood circulation and removing blood stasis)* has been advocated as a potential therapeutic approach [8, 9]. There are several reasons accounting for this theory. One is that cancer patients usually show *Xue-Yu ZHENG*. For example, patients with liver cancer usually exhibit bluish tendon on abdomen, scaly skin, a darkened complexion on the face, a hump below the costal region, and a purple-colored tongue and complain of a localized pricking pain in the region corresponding to the liver [10]. These symptoms are indicators of *Gan-Xue-Yu* (blood stasis in the *Gan*) and should be treated with the aim of *Huo-Xue-Hua-Yu* (as described above). Another reason is that cancer patients with *Xue-Yu ZHENG* usually present with microcirculation disturbance [11]. For example, Liu et al. observed that lung cancer patients with *Xue-Yu ZHENG* had significantly higher fibrinogen content than their counterparts without *Xue-Yu ZHENG*; moreover, the increased fibrinogen was found to be correlated with increased metastasis [8]. Another observational study from 105 patients with liver cancer demonstrated that the presence of *Xue-Yu ZHENG* was associated with a worse prognosis; it was unclear whether treatment for *Huo-Xue-Hua-Yu* in these patients significantly affected patient survival [12]. The third reason is that a tumor-mediated hypercoagulable state may exist and functionally complicate the disease state. The tumor-mediated hypercoagulable state is known to promote expression of tissue factor (TF) on the surfaces of tumor cells and macrophages, cell surface phospholipids that support coagulation activation, other tumor-mediated factors that trigger platelet activation and support accumulation, and tumor-induced endothelial cell factors that activate coagulation [13]. Furthermore, recently published preclinical data has suggested that activation of coagulation can promote tumor growth and angiogenesis. Since clinical hypercoagulable status is associated with adverse cancer prognosis, treatment with anticoagulation agents may prolong survival in certain types of cancer [14].

Even though a definitive link between cancer and *Xue-Yu ZHENG* has not yet been identified, some studies have shown evidence that *Huo-Xue-Hua-Yu* treatment may promote cancer metastasis. A prospective randomized controlled trial in 60 nasopharyngeal carcinoma cases conducted by Han et al. showed that integrated *Huo-Xue-Hua-Yu* herbs treatment with radiotherapy in nasopharyngeal carcinoma patients was associated with a 2.67-fold increase in distant metastasis, as compared to patients receiving radiotherapy alone [15]. In addition, preclinical studies showed that some *Huo-Xue-Hua-Yu* medicines, such as Danshen (*Red-rooted salvia root*), Chishao (*Red paeony root*), Danggui (*Chinese angelica*), Honghua (*Indian azalea leaf*), Jixueteng (*Suberect spatholobus stem*), Awei (*Chinese asafoetida*), and Chuanxiong (*Szechuan lovage rhizome*), could promote lung metastasis in liver cancer xenografted mouse models [16]. Our group previously established a xenograft tumor mouse model with *Xue-Yu ZHENG* to evaluate the effect of *Xue-Yu ZHENG* on tumor metastasis. We found that mice with the *Xue-Yu ZHENG* developed less metastasis than their counterparts without *Xue-Yu * [17–20]. However, when the tumor-bearing mice with *Xue-Yu ZHENG *were treated with individual *Huo-Xue-Hua-Yu* herbs, such as Danshen (*Red-rooted salvia root*) and Shensanqi (*Sanchi*), we found that Shensanqi treatment suppressed liver metastasis [19, 20] while Danshen treatment promoted liver metastasis [19]. Therefore, the correlation between *ZHENG* and cancer cells needs to be further studied in order to gain a more comprehensive understanding of its effects on the complex processes of tumor growth and metastasis. 

### 5.2. *Shi-Re ZHENG* (Dampness-Heat)

*Shi-Re ZHENG* is caused by dysfunction of the *Pi *(“spleen”) and *Wei *(“stomach”) due to retention of dampness and heat in the body. The occurrence of *Shi-Re* is usually based on water and wetness. The water and wetness can change into heat if they are stored in the body for long periods, and the combination of water and wetness and heat may cause *Shi-Re ZHENG*. *Shi-Re* is characterized by epigastric or abdominal oppression, lack of appetite, heavy body weight, thirst with little/no desire to drink, abdominal pain, loose stools, nausea, vomiting, fever, headache, red tongue body with a yellow sticky coat, and/or slippery rapid pulse.

*Shi-Re ZHENG* has been associated with many diseases, especially those involving the gastrointestinal (GI) tract. The potential molecular basis of *Shi-Re ZHENG* has attracted much research attention, although it is far from clear. Recently, *Shi-Re ZHENG* has been implicated in a broad range of inflammatory conditions, including eczema, psoriasis, cystitis, urethritis, gastroenteritis, vaginitis, cervicitis, meningitis, conjunctivitis, rheumatoid arthritis, and allergic reactions [21–25]. In addition, *Shi-Re ZHENG* was found to correlate with changes in expression of inflammation cytokines. For example, Liu and Wang showed that the serum levels of tumor necrosis factor-*α* (TNF-*α*) and interleukin-13 were significantly higher in rats with ulcerative colitis complicated with *Shi-Re ZHENG*, as compared with those without *Shi-Re* [26]. Likewise, Jiang et al. observed that in a total of 63 patients with chronic hepatitis B, 27 cases were diagnosed with *Shi-Re ZHENG*, and patients with *Shi-Re* had higher levels of TNF-*α* and tissue inhibitor of metalloproteinases (TIMP)-1 [27].

**Figure 3:**
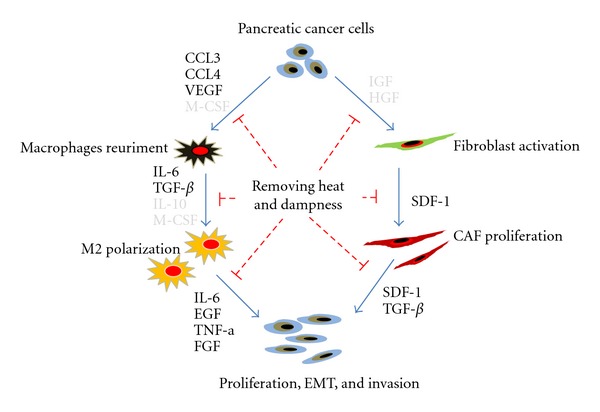
A schematic cartoon portraying the molecular basis of *Shi-Re ZHENG* in pancreatic cancer, based on our previous studies. It has been proposed that tumors with *Shi-Re ZHENG* exhibited altered cancer-associated myofibroblast (CAF) proliferative activities and tumor-associated macrophage (TAM) infiltration, which led to altered levels of CAF- and TAM-derived secreted cytokines (such as SDF-1, VEGF, TGF-1*β*, IL-6, CCL3, CCL4, CCL5, TNF-*α*, IL-8, and bFGF). The presence of *Shi-Re ZHENG* has impact on tumor growth. Chinese herbs for *Qing-Re-Hua-Shi* (removing heat and dampness) inhibited cancer cell proliferation, invasion, and *in vivo* metastasis through modification of the tumor microenvironment. Cytokines that are marked in bold have been confirmed by our previous studies.

It has been reported that symptoms of *Shi-Re ZHENG* are commonly seen in patients with GI cancer, including cancers of the duodenum, colon, liver, pancreas, and gallbladder. Just as we have shown in Figure 2, *Shi-Re* has been reported as one of the most common *ZHENGs* in liver, colorectal, and pancreatic cancers. However, there is still not a clear understanding of the biological validity of *Shi-Re* and the possible mechanisms of *ZHENG* in cancer. Our research group has previously established a pancreatic cancer xenograft mouse model with *Shi-Re ZHENG* [28]. We found that *Shi-Re ZHENG* mice exhibited altered cancer-associated myofibroblast (CAF) proliferative activities and tumor-associated macrophage (TAM) infiltration, which led to altered levels of CAF- and TAM-derived secreted cytokines (such as, SDF-1, VEGF, TGF-1*β*, IL-6, CCL3, CCL4, CCL5, TNF-*α*, IL-8, and bFGF). The presence of *Shi-Re ZHENG* has also been shown to impact tumor growth. Chinese herbs for *Qing-Re-Hua-Shi* (removing heat and dampness) were found to inhibit cancer cell proliferation through modification of the components of the tumor microenvironment [28–30]. These findings suggested that *Shi-Re* is associated with altered tumor microenvironment (Figure 3). 

### 5.3. *Yin-Xu ZHENG* (Yin Deficiency)

*Yin-Xu* represents insufficiency of body fluid. It is characterized by dryness in the throat and/or mouth, perspiration during sleep, tinnitus, dizziness, fatigue, insomnia, red tongue body with no coating on, and pulse that is thin, fine, or floating and empty. *Yin-Xu* may occur in many organs, including the stomach, lung, liver, kidney, or heart. Symptoms of *Yin-Xu ZHENG* are commonly seen in cancers of the liver, lung, breast, stomach, and colon (Figure 2). However, only a few publications in the literature have studied the molecular basis of *Yin-Xu*. Shen et al. observed that in patiens with lung cancer, *Yin-Xu* was correlated with changes in the cytokine expression profile [31–34]. They also showed that lung cancer tissue with *Yin-Xu ZHENG* exhibited dysregulated expression of TNF-*α*, IL-l*α*, IFN, IL-2, IL-8, and IL-1R*α*, as compared with that without *Yin-Xu ZHENG*. Thus, the molecular basis of *Yin-Xu ZHENG* is believed to involve components of the inflammatory cytokines network. 

### 5.4. *Pi-Xu* (Spleen Deficiency)

For this *ZHENG*, the word “spleen” does not refer to the organ, as in western medicine. It is a term used to describe an entire group of physiological functions. Based on the so-called *Pi-Wei theory* (also called *spleen-stomach theory)*, the *Pi* (“*spleen*”) governs molecular transport and transformation since the *Pi* transforms food into nutrients, which are the sources of *Qi* and blood, and distributes the nutrients to the limbs and other organs. Hence, the theory of “*Pi* being acquired foundation” has emerged. This theory postulates that when there is *Pi-Xu*, the digestion process is perturbed, causing abdominal discomfort and making the person feel tired.Since the *Pi* would normally keep the body fluids flowing in their respective pathways, signs of *Pi-Xu ZHENG *are hemorrhage, swelling, and bruising.

*Pi-Xu* has been shown to be involved with dysfunction of the vegetative nervous system of the GI tract, immune pathways, and endocrine processes. It can also mediate the distribution and content of fecal bacteria flora and gut-associated microbiota, including ulcer- and inflammation-causing *Helicobacter pylori*, as well as trace elements involved in blood and muscle metabolism [35]. Patients with different cancer types, in addition to the GI type, may present with *Pi-Xu* at various stages of the disease. Because many if not all cancers share at least some pathophysiological features, it is possible that they may be treated by an intervention approach based on a single principle but with flexibility to allow emphasis on different aspects of the disease in different patients. 

Extensive research has been carried out to determine the molecular basis of *Pi-Xu* in cancer. Since the 1960s, a group led by Yu Erxin has performed a series of investigations in liver cancer patients to investigate the potential molecular components of *Pi-Xu* [36, 37]. These efforts have identified a correlation between *Pi-Xu* and immunological dysfunction [38]. Liver cancer xenograft mice with *Pi-Xu* were shown to have significantly less total T cells and T helper (Th) cell lymphocytes, but more inhibitory T cells, than their counterparts without *Pi-Xu*. Furthermore, when these *Pi-Xu* mice were treated with Dangshen (*Pilose asiabell root*) and Huangqi (*Pilose asiabell root*) combination therapy, the level of Th cell-expressed CD4 was elevated significantly. Thus, it is believed that *Pi*-fortifying prescriptions may enhance proliferation of splenic cells and significantly increase auto-antibody secretory cell number, thereby enhancing the cytotoxic action of lymphocytes. Indeed, it has been shown that administration of *Pi*-fortifying therapy to ConA-stimulated mice promotes splenic cells to secrete cytokines, such as IL-2 [38]. Likewise, clinical observation in patents with liver cancer showed that patients with *Pi-Xu* were treated with *Pi*-fortifying therapy the activities of both natural killer cells and lymphokine-activated killer cells were restored [39]. 

*Pi-Xu* has been correlated with the abnormal energy metabolism that occurs in tumor cells. Observational study from 40 cased with liver cancer showed that liver cancer patients with *Pi-Xu* exhibited decreased serum levels of cyclic adenosine monophosphate (cAMP), while those patients with *Shi-Re* or *Xue-Yu* showed no significant changes in cAMP level [40]. Liver cancer xenograft mice with *Pi-Xu* also showed decreased serum and splenic cAMP levels, and increased cGMP and cAMP/cGMP ratio; intriguingly, the condition was not improved or resolved by treatment with the *Pi*-fortifying prescriptions [41]. These findings were also observed in patients with gastric cancer [42]. In addition to its effects on immune-related mechanisms and energic metabolism, the *Pi*-fortifying prescriptions was also shown to mediate the patterns of trace elements [43, 44]. Patients with various chronic diseases and *Pi-Xu* present with altered expression and distribution patterns of trace elements, including Cu, Zn, and Fe [45]. In gastric cancer patients with *Pi-Xu*, the levels of Cu and Zn are significantly changed, in particular [46]. Therefore, *Pi-Xu* is a now considered as a multisystem functional impairment. 

## 6. Prospects and Challenges

In TCM, the medicines are prescribed according to *ZHENG*, and *ZHENG* remains the essence of TCM treatment. However, there are some important issues that deserve mentioning. First, as TCM *ZHENG* differentiation is usually based upon the treating physician's intuition and personal experience, results differ from physician to physician and from clinic to clinic. Thus, *ZHENG* differentiation has a low reproducibility. To date, no unified criteria have been published for *ZHENG* differentiation, and it remains one of the main obstacles to widespread application of TCM in the clinical and research settings. Second, in this summary analysis, we emphasized the important position of *ZHENG* since it helps to guide the design of an individual's treatment regimen. We believe that the results of this study may help provide a theoretical basis for clinical diagnosis and treatment. However, we also recognize that when used as the sole treatment for cancer, TCM *ZHENG* does not consistently produce satisfactory therapeutic efficacy. Recently, there has been much interest in the potential clinical utility of “analogous *ZHENG* existing in the same disease” for improving TCM in clinical practice [47], especially for cancer patients. Thus, a strategy combining *ZHENG* differentiation and disease diagnosis is considered promising for future cancer treatment.

While much research has attempted to elucidate the molecular basis of the cancer-associated *ZHENGs*, the available data are subject to several limitations that must be considered when contemplating the utility of TCM *ZHENG *as a cancer therapy. First, TCM is focused on alleviating a particular disease or condition, while the *ZHENG* is based on systemic and holistic concepts. Therefore, a system's biology approach may be the optimal way to research the clinical utility and therapeutic efficacy of TCM *ZHENG*. Second, TCM is practiced with respect to the rules of “treating the same disease with different methods” and “treating different diseases with the same methods”. In our summary analysis, we found the same molecular basis underlying the same *ZHENG* in different diseases. However, we should also emphasize that molecular differences that are disease- or diagnosis-specific, while sharing a *ZHENG*, may prove particularly important in designing effective individualized treatment regimens. This notion is consistent with the current understanding that combination of *ZHENG* differentiation and disease diagnosis yields improved treatment efficacy. Third, we point out that a comprehensive profile of *ZHENG*-specific molecules has yet to be identified, and the correlation between *ZHENG* and molecules has yet to be firmly established. Finally, it is important to remember that *ZHENG* is now considered as a multisystem and multiorgan functional impairment. Although modern technologies have been applied to *ZHENG* research, we are far from obtaining a clear understanding of the exact molecular basis of *ZHENG*. We are hopeful that future integration of modern technologies and continued research may eventually promote *ZHENG* research.

## 7. Conclusions

In this study, we systematically identified the collected body of research on TCM *ZHENG* in cancer patients. The sources of these data were the publically available Chinese language scientific and medical literature databases. We first summarized the clinical *ZHENG* distribution among six common cancer types, including lung, liver, gastric, breast, colorectal, and pancreatic, which may help to provide a theoretical basis for TCM as a clinical cancer treatment. We then considered the molecular basis of *Xue-Yu*, *Shi-Re*, *Yin-Xu*, and *Pi-Xu ZHENGs* that are commonly present in different types of cancer, which may contribute to a better understanding of the potential of TCM *ZHENG* for supplementing modern therapeutic strategies for cancer.
